# Supplementary material for: Sablefish (Anoplopoma fimbra Pallas, 1814) plasma biochemistry and hematology reference intervals including blood cell morphology
Source: PLoS One. 2021 Jun 4;16(6):e0246982. doi: 10.1371/journal.pone.0246982 (PMC8177537; doi:10.1371/journal.pone.0246982)

Sablefish (*Anoplopoma fimbria*) plasma biochemistry and hematology reference intervals including blood cell morphology

Carla B. Schubiger^1*^, M. Elena Gorman^1^, Jennifer L. Johns^1^, Mary R. Arkoosh^2^, Joseph P. Dietrich^2^

^1^Carlson College of Veterinary Medicine, Oregon State University, Corvallis, Oregon, USA

^2^Environmental & Fisheries Science Division, Northwest Fisheries Science Center, National Marine Fisheries Service, National Oceanic and Atmospheric Administration, Newport, Oregon, USA

*Corresponding author: Carla.schubiger@oregonstate.edu

**S1 Figure.**


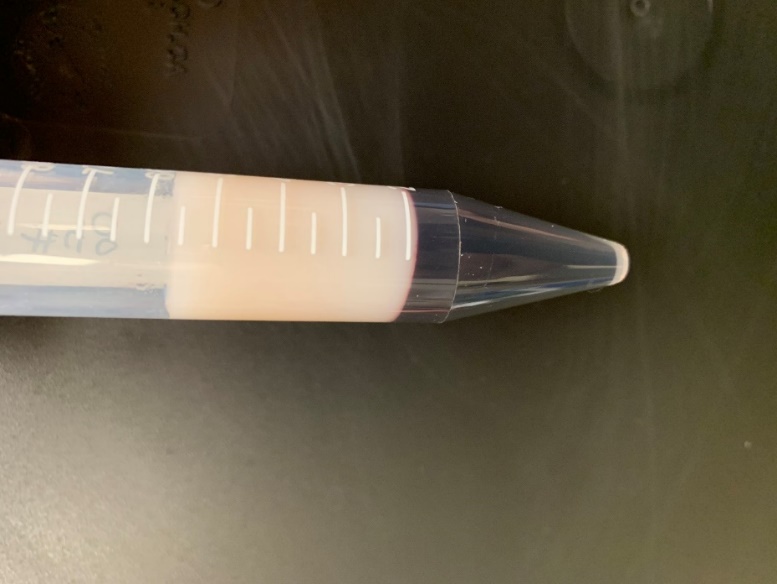

Supplement: S1 Fig — Photograph of lipemic blood sample from fish #34 on June 20th, 2019, after centrifugation. A packed cell fraction (bottom of tube) is overlaid by a turbid, pale-beige plasma. The turbidity indicates a significant amount of blood lipids. (DOCX) [file pone.0246982.s001.docx]
